# Efficacy of Vitamin D_3_ Buccal Spray Supplementation Compared to Other Delivery Methods: A Systematic Review of Superiority Randomized Controlled Trials

**DOI:** 10.3390/nu12030691

**Published:** 2020-03-04

**Authors:** Maria G. Grammatikopoulou, Konstantinos Gkiouras, Meletios P. Nigdelis, Dimitrios P. Bogdanos, Dimitrios G. Goulis

**Affiliations:** 1 Department of Rheumatology and Clinical Immunology, Faculty of Medicine, School of Health Sciences, University of Thessaly, Larissa GR41110, Greece; kostasgkiouras@hotmail.com (K.G.); bogdanos@med.uth.gr (D.P.B.); 2 Laboratory of Clinical Pharmacology, Medical School, University Campus, Aristotle University of Thessaloniki, Thessaloniki GR54124, Greece; 3 Unit of Reproductive Endocrinology, 1st Department of Obstetrics and Gynecology, Medical School, Aristotle University of Thessaloniki, Thessaloniki GR56429, Greece; meletis.nigdelis@gmail.com (M.P.N.); dgg@auth.gr (D.G.G.); 4 Division of Transplantation, Immunology and Mucosal Biology, MRC Centre for Transplantation, King′s College London Medical School, London SE5 9RS, UK

**Keywords:** vitamin D, cholecalciferol, dietary supplement, oral spray, sublingual spray, oral drops, capsules

## Abstract

(1) Background: Vitamin D deficiency is an important public health concern and supplementation is common for this deficiency. Many different modes of delivering supplementation have been proposed in order to enhance absorption and utilization. The present review compared the efficacy of vitamin D_3_ buccal spray against other forms of supplementation delivery. (2) Methods: The protocol was registered at PROSPERO (CRD42019136146). Medline/PubMed, CENTRAL and clinicaltrials.gov were searched from their inception until September 2019, for randomized controlled trials (RCTs) that compare vitamin D_3_ delivery via sublingual spray against other delivery methods. Eligible RCTs involved humans, of any age and health status, published in any language that evaluated changes in plasma 25(OH)D concentrations. Three reviewers independently extracted data, assessed risk of bias (RoB) and the quality of the trials. (3) Results: Out of 9759 RCTs, four matched the predefined criteria. Intervention duration ranged from 30 days to 3 months whereas vitamin D_3_ dosage ranged between 800 and 3000 IU/day. One RCT advocated for the superiority of buccal spray in increasing plasma 25(OH)D concentrations, although several limitations were recorded in that trial. The rest failed to report differences in post-intervention 25(OH)D concentrations between delivery methods. Considerable clinical heterogeneity was observed due to study design, intervention duration and dosage, assays and labs used to perform the assays, population age and health status, not allowing for synthesis of the results. (4) Conclusions: Based on the available evidence, delivery of vitamin D_3_ via buccal spray does not appear superior to the other modes of delivery. Future RCTs avoiding the existing methodological shortcomings are warranted.

## 1. Introduction

Vitamin D_3_ is an essential fat-soluble nutrient involved in a plethora of metabolic pathways [1,2,3,4,5]. When consumed within the dietary reference level limits, vitamin D exerts multiple health benefits [6,7,8]. Apart from food sources, the majority of vitamin D_3_ is produced non-enzymatically via ultraviolet-B (UVB) exposure of 7-dehydrocholesterol on skin [6,9,10,11]. Despite the existence of this additional pathway to increase plasma 25-hydroxycholecalciferol (25(OH)D) concentrations, vitamin D deficiency remains an important global challenge [12,13,14] with supplementation being proposed for several conditions including pregnancy [15,16,17,18,19], ageing [20,21], obesity [22,23,24,25], infertility [26,27], skeletal health [28], glycemic control [29] and diabetes [30,31], abnormal lipidemic profile [32], cardiovascular [33,34], autoimmune [35,36,37,38,39] and liver disease [40,41].

Determining vitamin D status based on serum 25(OH)D levels remains controversial [11], with some agencies suggesting at least 75 nmol/L to ensure replete status [42], whereas others recommend >50 nmol/L [43,44]. Maintaining a serum 25(OH)D concentration above 50 nmol/L is considered as optimum according to some organizations [43,44], with others advocating against the development of severe deficiency (defined as 25(OH)D < 25 nmol/L) [45]. Given the importance of the vitamin and the relatively frequent shortfall observed in most populations, a variety of recommendations exist [11]. Currently, breastfed infants are required to consume 400 IU daily, and thereafter, an amount of 400–800 IU is recommended each day throughout the life cycle [11,43,44,46], with the exception of the Endocrine Society Guidelines [42], which suggest an even greater upper threshold regarding the reference intake, especially during pregnancy and lactation. Recommendations concerning supplementation dosage and duration remain heterogenous, based on geographical latitude, sun exposure, age, skin phenotype, diagnosed comorbidities that alter vitamin D metabolism, as well as vitamin D and weight status [11].

Apart from supplementation frequency [47], it has been suggested that the mode of supplementary vitamin delivery affects bioavailability, release, and absorption, as well as unstable compounds decomposition [12,48,49]. Subsequently, aside from the typical soft capsule form, several novel delivery methods have been proposed, including gels, oral drops, gums [50] and more recently, sublingual buccal spray [51,52].

Individual randomized controlled trials (RCTs) on the efficacy of vitamin D_3_ buccal spray suggest its superiority against other modes of delivery [51]. However, given that buccal sprays are approximately double the price of the commonly prescribed capsules, the need for meta-research of the current evidence is vital for consumers and health insurance companies.

Therefore, the aim of the present study was to systematically review individual RCTs that assess the superiority of buccal spray against other modes of vitamin D_3_ delivery.

## 2. Materials and Methods

### 2.1. Search Strategy

**Figure 1:**
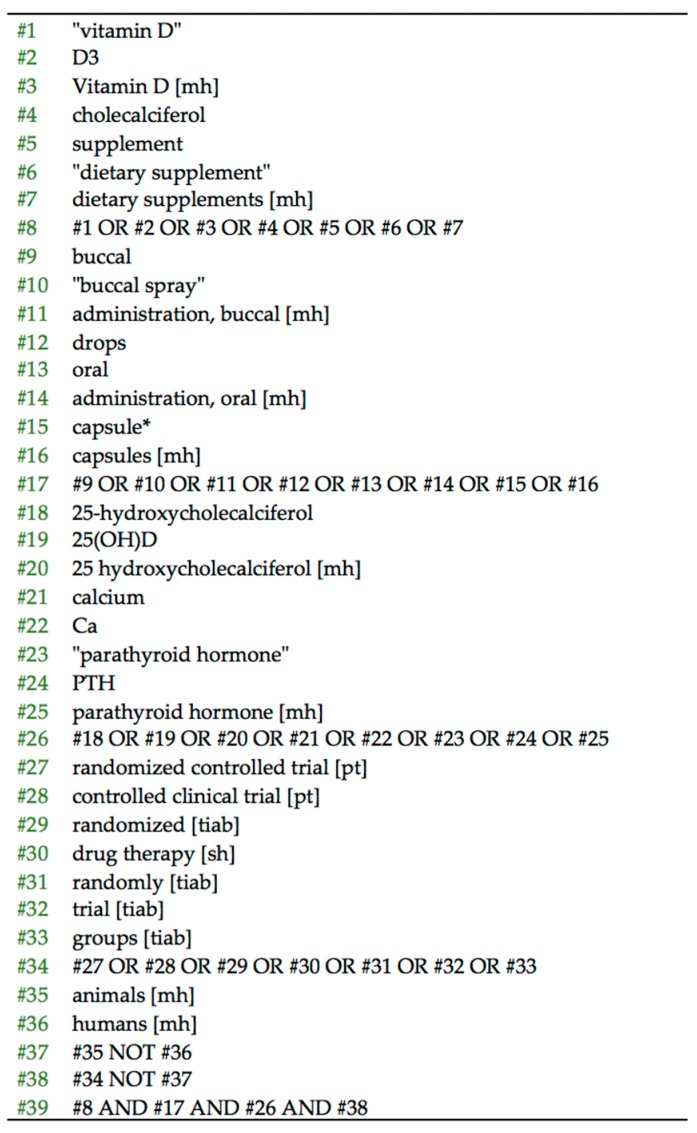
PubMed search strategy.

The protocol was registered at PROSPERO (CRD42019136146) and OSF. A comprehensive search was performed in PubMed, Cochrane CENTRAL, and ClinicalTrials.gov for RCTs comparing vitamin D_3_ supplementation via buccal spray against other delivery methods, from the site’s inception until September 2019. Table 1 summarizes the PICO (Population - Intervention - Comparison - Outcome) [53] strategy applied to the study’s research question. The keywords used were vitamin D, vitamin D_3_, cholecalciferol, 25-hydroxycholecalciferol, dietary supplement, buccal spray, oral, drops, administration, calcium, parathyroid hormone, with a combination of medical subheadings (MeSH) terms when applicable. A detailed PubMed search strategy is presented in Figure 1.

### 2.2. Search Eligibility Criteria

Inclusion criteria were RCTs on (1) any population, including healthy participants or patients, (2) populations with low serum 25(OH)D concentrations, (3) of any age group, (4) living in any country, (5) comparing vitamin D_3_ buccal spray against other routes of vitamin D_3_ delivery (i.e., oral drops, capsules), (6) performed on humans, (7) using any RCT design, (8) published in any language.

Exclusion criteria involved (1) non-randomized trials, (2) comparing vitamin D_3_ buccal spray against placebo, or (3) studies performed on animals.

### 2.3. Selection of Studies and Interventions of Interest

Initially, three independent reviewers (M.G.G., K.G. and M.P.N.) identified studies from their titles and abstracts. Full-text articles were retrieved to assist decision-making in cases when deemed necessary. Any disagreement between reviewers was resolved by a senior researcher (D.G.G.).

### 2.4. Data Exctraction

Two reviewers (M.G.G. and M.P.N.) independently extracted characteristics of the retrieved RCTs and outcomes of interest from full-text articles. Extracted data involved (1) the number of participants at each stage, (2) participant characteristics, (3) study characteristics (registry, design, ethical approval, country, funding), (4) administered dose of vitamin D3 and methods of delivery, (5) intervention duration, (6) washout period (whenever applicable), (7) participant recruitment sites, (8) assays and kits for determining 25(OH)D levels, (9) baseline and post-intervention results (including 25(OH)D, Ca, and parathyroid hormone (PTH) concentrations), (10) recorded adverse events, (11) drop-outs, and (12) analysis performed (intention-to-treat or per protocol).

Data were extracted using a predefined Microsoft Excel data extraction form, including study (design, funding, allocation concealment, protocol registry, country, recruitment site) and participant characteristics (age, health conditions, discontinued/dropouts), intervention details (form, duration, dosage, adverse events), comparators, and clinical outcomes to produce an overview table of all eligible studies.

Characteristics of the retrieved RCTs were evaluated with the Cochrane risk of bias (RoB) 2.0 tool [54] by two reviewers (M.P.N. and M.G.G.) independently, in order to present bias comprehensively. A more experienced author (D.P.B.) assessed between-reviewer differences. The RoB results classified studies as being of “high”, “unclear” or “low” risk of bias. Additionally, the Oxford quality scoring system (Jadad score) [55] was applied on each RCT to assess trial quality.

## 3. Results

### 3.1. Study Selection

**Figure 2:**
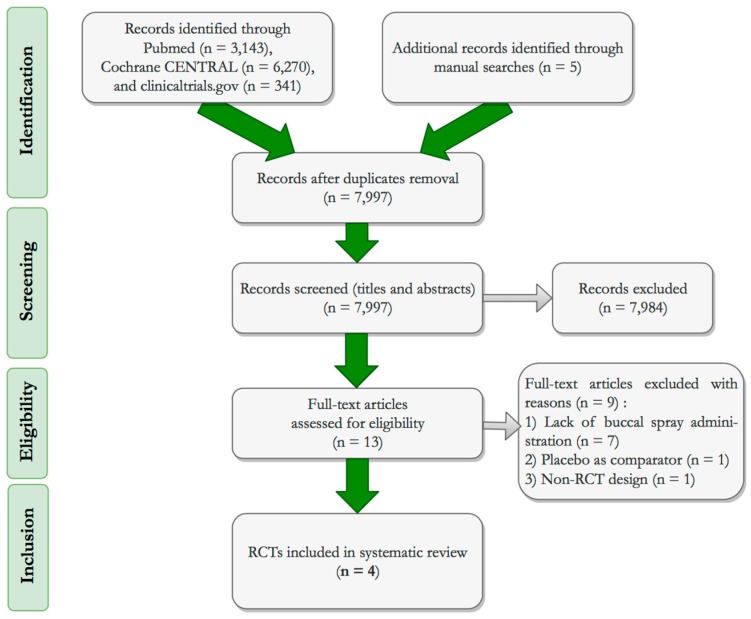
PRISMA flowchart [56] of the studies selection process.

A total of 9759 studies were screened by title and abstract and 13 were assessed for eligibility criteria (full-text screening), out of which nine were excluded for having a different mode of supplementation delivery, comparing against placebo, or lacking a RCT design. The PRISMA flowchart [39] was applied to illustrate the step-by-step exclusion of unrelated/duplicate retrieved records, leading to the final selection of four RCTs that met the predefined inclusion criteria (Figure 2).

Table 2 summarizes the characteristics of the retrieved RCTs. Two trials [51,57] had a crossover design, and the remaining two [58,59] used parallel interventions. One RCT was multicenter [51] and single-blinded. The rest were single-center, two of which used open-label [57,58] and one used double-blind masking [59]. Intervention duration ranged between 30 days to 3 months and was mainly performed during winter time. Only one RCT [59] evaluated participants’ skin tone during the study. Vitamin D_3_ dosage ranged from 800 [58] to 3000 [57,59] IU per day. As far as participants are concerned, Satia [51], Todd [57] and Williams [59] used adult samples, whereas Penagini [58] recruited children with neuro-disabilities. On the other hand, Satia [51] included two participant arms, one consisting of healthy subjects, and the other comprising patients with malabsorption syndrome. The Todd [57] trial was restricted to the recruitment of healthy adults.

Satia [51] and Williams [59] also compared against a placebo, but these comparisons were omitted from the present analyses for not fulfilling the “superiority” comparison criterion.

### 3.2. Risk of Bias and Quality Assessment of Studies

The risk of bias of the included studies is illustrated in Table 3. The Penagini [58] trial was assessed as having a high-risk of overall bias, for lacking a predefined protocol, randomization, and funding disclosure. Williams and associates [59] also conducted a trial of high overall bias, given that the predefined intervention duration was not kept. The RCT by Satia [51] was of unclear bias, with substantial deviations from the reported intended interventions.

Quality assessment of the RCTs based on the Jadad [55] scale (Table 2) revealed that the Satia [51] and Todd [57] trials exhibited several bias-related issues. On the other hand, the RCT performed by Penagini [58] demonstrated the most quality issues, including bias in the randomization process, deviations from the intended interventions, overall bias and unclear risk outcomes measurement and selective reporting. In contrast, the study conducted by Williams [59] received the highest quality score among all of the studies. Additionally, Todd [57] failed to separate the intention-to-treat from the per-protocol analyses, whereas Penagini [58] and Williams [59] lacked many of the CONSORT [60] components, including a flow diagram or details concerning dropouts and the number of participants at each stage. None of the RCTs reported any post-intervention adverse event, except Williams et al. [59] who reported small blisters on the cheek and tongue of two participants.

Satia [51] was the only one who advocated for the superiority of vitamin D_3_ buccal spray against the other modes of delivery in increasing plasma 25(OH)D concentrations. The remaining three RCTs [57,58,59] did not report any difference between intervention and comparator groups, and indicated the similarity and equal efficacy between different modes of vitamin D_3_ delivery.

## 4. Discussion

Although a variety of delivery methods exist for most dietary supplements, systematic reviews and meta-analyses on the efficacy of each mode are lacking. The present systematic review indicates that vitamin D_3_ delivery via buccal spray does not differ from other supplementation methods in increasing plasma 25(OH)D levels. In parallel, the small number of retrieved RCTs and the high degree of clinical heterogeneity among them did not allow for a safe synthesis of the results as initially intended.

The Satia [51] trial was the only one that reported positive findings regarding the superiority of vitamin D_3_ delivery via buccal spray compared to capsules. However, the trial has limitations regarding the washout duration. According to Senn [61], if the duration of the washout is reasonable, substantial carry-over effects are unlikely to occur. On the other hand, as Todd and associates [57] note, the washout duration must be based on the US Food and Drug Administration (FDA) rule of thumb [62,63], which is five times the plasma half-life of the measured substance, herein 25(OH)D, is needed to achieve elimination of more than 95% of the substance from the body. Given that the plasma half-life of total 25(OH)D is approximately 15 days [64], ten weeks are needed to wash out any supplementation effect. Thus, based on the FDA guidelines, the duration of washout carried out by Satia [51] (10 days) appears inadequate.

According to the literature, interpretation of vitamin D assay results should be performed with caution, as not all methods are equal [65]. Farrell [66] revealed that automated immunoassays tend to demonstrate variable performance, and often fail to meet specific performance goals. On the other hand, the liquid chromatography-tandem mass spectrometry (LC-MS/MS) method used by Todd [57] tends to exhibit greater accuracy, lower variability and less bias [65,66]. Apart from the distinct assays, all three trials used independent laboratories and this has been shown to produce further variations in the results [67], as most laboratories fail to adhere to quality assurance standards and comply with international standardization processes.

Apart from the low Jadad [55] score and high risk of bias, the Penagini [58] trial demonstrated several additional shortcomings. Except for supplements, two known physiological pathways exist for increasing 25(OH)D concentrations, with the first being epidermal synthesis via sun exposure and the second through dietary intake. Concerning the latter, several studies suggest that vitamin D absorption is enhanced with concomitant fat intake or other oily vehicles [68]. Penagini [58] did not report controlling for these factors, failed to state the season in which the intervention was implemented and to include the assessment of usual dietary vitamin D intake, which introduces possible bias in the trial results. According to Rees [69], lifestyle variations account for one half of the variability in vitamin D supplementation response; thus, all trials should adjust for these factors in advance.

An additional limitation of the included RCTs is the lack of vitamin D genetic variants assay. As with most procedures in the human body, vitamin D absorption and utilization are also epiphenomena related to hereditary susceptibility, which suggests a personalized response [10,70]. Hence, genetic variations in 25-hydroxylase and vitamin D-binding protein have been shown to alter supplementation response [71,72], although the produced effect appears small compared to that of lifestyle components [69]. However, none of the included RCTs reported assessing vitamin D genetic variants or controlling for them during sample recruitment and group allocation.

Taking into account all of the above issues, the clinical heterogeneity of the retrieved RCTs appears to be multifactorial, which stems from the different study design, assays and laboratories used to perform the assays, intervention dosage, duration and season, washout duration, participant age and health status, allocation concealment and usual dietary intake. Although individually these factors are often encountered in meta-analyses, when only four trials are concerned, the coexistence of all these factors exacerbates heterogeneity and does not allow for a safe synthesis of the results. Indeed, in an attempt to pool findings (K.G.), considerable statistical heterogeneity was observed; thus, we considered that based on the currently available evidence at this time, a systematic review would be more robust compared to a meta-analysis.

Secondary analyses and synthesis of the findings of trials assessing the efficacy of vitamin D supplementation are required to produce robust results [73]. To this point, there are no other published systematic reviews that evaluate different modes of delivering dietary supplements. The present review was structured to assess the efficacy of vitamin D_3_ supplementation from a different point of view: the superiority of buccal spray mode of delivery. Of note, one protocol for a systematic review with some similar features was published approximately a year ago (CRD42018118580) [74], although no preliminary or final findings have been reported until now. Distinct differences exist between the two protocols, with the present one focusing solely on vitamin D_3_, using a RCT design as an inclusion criterion, while assessing any form of vitamin D_3_ oral spray supplementation delivery. On the other hand, the other protocol [74] reported the inclusion of any quasi-experimental study, focuses on both vitamin D_2_ and D_3_ intervention studies, while excluding spray interventions applied to the buccal mucosa, as performed in the Williams [59] trial included herein (use of sublingual spray). Additionally, a variety of methodological differences can be observed, including the search strategy, databases, search strings and keyword combinations applied, the tools used for assessing the quality and bias of studies (with the Jadad [55] and RoB 2.0 [54] being used herein, compared to the Joanna Briggs Institute (JBI) and GRADE [75] applied in the other protocol), and distinct data extraction protocols. In comparison to the aforementioned protocol [74], the present review has more restrictions with regard to the search strategy, as well as concerning the eligibility criteria, narrowing down the results to a great extent, while differentiating primary outcomes synthesis. Subsequently, based on the distinct methodological designs, inclusion/exclusion criteria, search strategy and vitamin D form based on the reported PICOs, the two studies would be expected to retrieve different primary studies, resulting in distinctive findings overall. Saldanha [76] noted that even when similar interventions are compared in trials or systematic reviews, differences in perspectives, goals, and constraints between trialists and reviewers explain differences in the outcomes. Nevertheless, as in primary research, also in meta-research, studies addressing similar research questions are required to inform practice and produce more robust recommendations. Given that 67% of the published meta-analyses tend to have at least one other overlapping meta-analysis, with a median of two meta-analyses per topic [77], and the fact that many differences exist between the two protocols, the two systematic reviews are expected to yield different findings based on a distinct qualitative synthesis of primary studies and are both required.

## 5. Conclusions

Thorough examination and critical appraisal of the current evidence reveals that despite the higher economic cost of the buccal spray, it does not appear to be superior to the other modes of vitamin D_3_ delivery. More RCTs are required to investigate its efficacy in distinct populations, including patients with malabsorption problems. The limitations of the existing trials highlighted herein could serve as a primer for the design of future, relevant RCTs in order to reduce heterogeneity, increase trial comparability, and increase the validity of individual RCT results. Nevertheless, vitamin D_3_ delivery via buccal spray might be preferred by populations with swallowing problems, or those receiving a great variety of supplements and/or medications, who wish to limit their intake of pills and capsules.

## Figures and Tables

**Table 1:** PICO strategy for the search question.

PICO	Description
**P**opulation	Any population, healthy or not
**I**ntervention	Vitamin D_3_ buccal spray supplementation
**C**omparison	Other modes of vitamin D_3_ supplementation delivery (capsules, drops, etc.)
**O**utcome	Change in serum 25(OH)D concentrations

*25(OH)D*: 25-hydroxycholecalciferol.

**Table 2:** Characteristics of the included randomized controlled trials.

First Author:	Satia [51]	Todd [57]	Penagini [58]	Williams [59]
Implementation year:	NR	2015–2016	2015–2016	2017
Publication year:	2015	2016	2017	2019
Design:	Cross-over	Cross-over	Parallel	Parallel
Masking:	Single-blinded	Open-label	Open-label	Double-blind
Multicenter:	√	-	-	-
Origin:	India	U.K.	Italy	UK
Registry:	CTRI/2013/06/003770	NCT02608164	NR	NR
Funding:	(1) Buccal spray provided by Pharma Base SA.	(1) Dept of Employment & Learning, N. Ireland (2) Translational Research Group, Public Health Agency, Belfast (3) Buccal spray provided by BetterYou Ltd.	NR	(1) BetterYou Ltd. (2) University of Sheffield
Ethical approval:	Spandan–Ethics	University of Ulster	University of Milan	University of Sheffield
Participant recruitment:	Two different hospitals, one physician’s site (healthy subjects) and a gastroenterologist’s site (patients with intestinal malabsorption)	The university and local area through circular emails and online advertisements	V. Buzzi Children’s Hospital	University of Sheffield
Participants (*n*):	N = 40 (healthy subjects and patients with malabsorption syndrome, ♂/♀ ratio = 1) Patients *n* = 14 ^‡^ Healthy controls *n* = 14 ^‡^	N = 22 healthy adults (♀ = 12)	N = 24 children (5–17 years old, ♀ = 14, with neuro-disabilities and vitamin D deficiency (cerebral palsy *n* = 7, symptomatic or genetic epilepsy *n* = 5, epileptic encephalopathy *n* = 9, genetic syndromes *n* = 3)	N = 50 ^¥^ non-obese, apparently healthy adults (18–50 years old, ♀ = 29)
Participant age (years):	Patients: 39.9 ± 11.7 Healthy controls: 36.2 ± 10	25.2 ± 6.5	Intervention: 7.8 (5–17) ^†^ Comparator: 9.4 (7–16) ^†^	Intervention: 21.7 ± 3.1 Comparator: 22.9 ± 4.8
BMI (kg/m^2^):	Patients: 21.5 ± 2.8 Healthy controls: 23.4 ± 3.9	Intervention: 24.2 ± 3.5 ^§^ Comparator: 24.4 ± 3.6 ^§^	Intervention: 18.2 (12.5–25.5) ^†^ Comparator: 16.9 (11.8–24.6) ^†^	Intervention: 23.8 ± 2.6 Comparator: 23.6 ± 3
Participant Groups (*n*):	Healthy participants: *n* = 14 ^*^ Patients: *n* = 14 ^*^	Intervention: *n* = 22 Comparator: *n* = 22	Intervention: *n* = 12 (♀ = 7) Comparator: *n* = 12 (♀ = 7)	Intervention: *n* = 25 (♀ = 15) Comparator: *n* = 25 (♀ = 14) (1) Active caps + placebo spray: *n* = 25 (2) Active spray + placebo caps: *n* = 25 (3) Double placebo: *n* = 25
Randomization:	Block, by statistician	MINIM software	NR	Block (size of 9), computer-generated
Vitamin D status definition:	Νone	Clinical deficiency: 25(OH)D < 30 nmol/L Insufficiency: 25(OH)D 31–49 nmol/L Sufficiency: 25(OH)D > 50 nmol/L	Deficiency: 25(OH)D ≤ 20 ng/mL	Deficiency: 25(OH)D < 30 nmol/L Insufficiency: 25(OH)D 31–46 nmol/L Sufficiency: 25(OH)D > 50 mmol/L ^ˆ^
25(OH)D assay:	ECLIA	LC-MS/MS	Immunoassay	LC-MS
Kit:	Roche diagnostics (GmbH, Germany)	API 4000; AB SCIEX, Chromsystems Instruments and Mass-Chrom 25-OH vitamin D_3_/D_2_; Chromsystems Instruments & Chemicals (GmbH)	25-Hydroxy Vitamin D EIA, Immunodiagnostic System, Ltd.	finger-prick blood spot
Assay laboratory:	Independent lab (APL Institute of Clinical Laboratory & Research Pvt. Ltd., Ahmedabad, IN)	Independent lab (Biochemistry Dept of St. James’ Hospital, Dublin, IE)	Pediatric Endocrinology Lab, Division of Genetics and Cell Biology, IRCCS San Raffaele Scientific Institute, Milan, IT	City Assays, Department of Pathology, Birmingham Sand-well Hospitals NHS Trust, UK
Exclusion criteria:	√	√	√	√
Intervention:	Buccal spray 2 shots x 500 IU vitamin D_3_/d	Buccal spray 3000 IU/d (75 μg) vitamin D_3_	Buccal spray 800 IU/d vitamin D_3_	Active vitamin D_3_ buccal spray 3000 IU (75 μg) + placebo caps
Comparators:	(1) soft caps (1000 IU) vitamin D_3_/d (2) none	3 × 1000 IU (25 μg) vitamin D_3_ caps/d, with water	Oral drops 750 IU/d vitamin D_3_	Active vitamin D_3_ caps 3000 IU (75 μg) + placebo spray
Intervention duration:	30 days	4 weeks	3 months	6 weeks
Season:	NR	Winter	Winter	Spring
Skin-tone evaluation:	NR	NR	NR	√
Washout duration:	30 days	10 weeks	NR	NR
Compliance assessment:	√	√	√	√
Dietary intake:	Recorded at baseline	Recorded at baseline	NR	NR
Analyses:	PP	ITT and PP	NR	ITT
Outcomes:	Δ in 25(OH)D levels	Δ in levels of 25(OH)D, creatinine, PTH, Ca, eGFR	Δ in levels of 25(OH)D, Ca, P, PTH, BAP, CTx	Δ in 25(OH)D levels
Dropouts:	*n* = 2 (low compliance)	*n* = 4 (3 went for a sun holiday, no longer wished to participate and 1 had illness unrelated to the intervention)	NR (flowchart lacking)	NR (flowchart lacking) *n* = 1 stopped due to adverse events without information on the allocation group
Baseline data (intervention group):	Healthy subjects: 18.9 ± 4.3 ng/mL (*n* = 13) Patients: 10 ± 4.3 ng/mL (*n* = 13)	25(OH)D: 59.6 ± 24.4 nmol/L (*n* = 22) Dietary vitamin D intake: 6.3 ± 6.2 μg/d PTH: 50.1 ± 26 pg/mL (*n* = 22) Ca: 2.2 ± 0.1 mmol/L (*n* = 22)	25(OH)D: 15.5 (8–20) ^†^ ng/mL PTH: 72.5 (31.4–145.8) ^†^ pg/mL Ca: 9.6 (9.1–9.8) ^†^ mg/dL	25(OH)D: 54.9 ± 27.8 nmol/L (*n* = 25)
Baseline data (comparator group):	Healthy subjects: 18.7 ± 5.9 ng/mL (*n* = 13) Patients: 11 ± 6.4 ng/mL (*n* = 13)	25(OH)D: 60 ± 26.3 nmol/L (*n* = 22) PTH: 50.3 ± 25.5 pg/mL (*n* = 22) Ca: 2.2 ± 0.1 mmol/L (*n* = 22)	25(OH)D: 11.5 (8–19) ^†^ ng/mL PTH: 65.9 (46–98.8) ^†^ pg/mL Ca: 9.4 (8.9–10.4) ^†^ mg/dL	25(OH)D: 50.7 ± 19.7 nmol/L (*n* = 25)
Results (intervention group):	Healthy subjects: 26.9 ± 5.7 ng/mL (*n* = 13) Patients: 20.5 ± 7.9 ng/mL (*n* = 13)	25(OH)D: 85.8 ± 19.4 nmol/L (*n* = 22) PTH: 48.2 ± 27.3 pg/mL (*n* = 22) Ca: 2.2 ± 0.1 mmol/L (*n* = 22)	25(OH)D: 26.5 (13.6–39) ^†^ ng/mL PTH: 48.9 (23.2–89.6) ^†^ Ca: 9.27 (8.7–10) ^†^ mg/dL	25(OH)D: 95.8 ± 28.0 nmol/L (*n* = 25)
Results (comparator group):	Healthy subjects: 22.8 ± 6.8 ng/mL (*n* = 13) Patients: 15 ± 9 ng/mL (*n* = 13)	25(OH)D: 90.4 ± 21 nmol/L (*n* = 22) PTH: 52.2 ± 19.3 pg/mL (*n* = 22) Ca: 2.2 ± 0.1 mmol/L (*n* = 22)	25(OH)D: 34.5 (22–49) ^†^ ng/mL PTH: 53.5 (30.6–98.4) ^†^ pg/mL Ca: 9.19 (8.6–9.8) ^†^ mg/dL	25(OH)D: 91.4 ± 19.8 nmol/L (*n* = 25)
Results overall:	The buccal spray significantly increased serum 25(OH)D levels as compared to the caps, in both healthy subjects and patients with malabsorption syndrome	No difference between buccal spray and caps	Vitamin D_3_ supplementation with buccal spray and oral drops are equally effective	Vitamin D_3_ supplementation via capsules and sublingual spray are equally effective
Adverse events:	NR	NR	NR	*n* = 2 small blisters on cheek and tongue
RCT Issues:	NR	NR	The dosage could not be matched precisely between the two interventions.	Dose inconsistency: The spray/caps content was prepared to 97.5 μg/dose in order to maintain shelf life and guarantee dose, however, each capsule and spray contained 3000 IU (75 μg) of vitamin D_3_ per dose.
Manuscript issues:	-	ITT and PP were not separated	No flowchart, no detailed *n* in each stage	No flowchart
Jadad [55] score:	2	2	−1	4

*BAP:* bone-specific alkaline phosphatase; *BMI:* Body Mass Index; *Ca:* Calcium; *CTRI:* Clinical Trial Registry India; *CTx:* C-terminal telopeptide of type I collagen; *ECLIA:* Electrochemiluminescence; *eGFR:* estimated glomerular filtration rate; *ITT:* Intention to treat; *IU:* international units; *LC-MS/MS:* liquid chromatography-tandem mass spectrometry; *NR:* Not Reported; *PP:* Per Protocol; *PTH:* Parathyroid Hormone; *RCT:* Randomized Controlled Trial; *25(OH)D:* 25-hydroxycholecalciferol. ^*^ Same *n* in intervention and comparator treatments for either group; ^†^ Expressed as median (range); ^‡^ Total *n* was 20 in each group, but the second comparator (placebo) was omitted from the present analyses; ^¥^ The second comparator (placebo), was omitted from the present analyses (*n* = 25); ^§^ At initial allocation, as this was a cross-over study; ^ˆ^ The mmol/l reported appears to be a typo and should probably be nmol/L.

**Table 3:** Summary risk of bias [54] assessment of the included randomized controlled trials.

	Randomization Process	Deviations from Intended Interventions	Missing Outcome Data	Measurement of the Outcome	Selection of the Reported Result	Overall Bias
Satia [51]	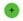	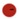	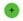	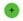	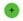	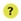
Todd [57]	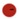	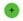	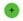	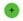	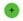	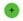
Penagini [58]	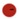	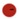	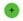	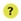	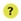	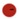
Williams [59]	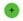	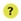	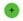	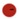	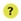	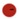
